# Long Term Dietary Restriction of Advanced Glycation End-Products (AGEs) in Older Adults with Type 2 Diabetes Is Feasible and Efficacious-Results from a Pilot RCT

**DOI:** 10.3390/nu12103143

**Published:** 2020-10-15

**Authors:** Roni Lotan, Ithamar Ganmore, Shahar Shelly, Moran Zacharia, Jaime Uribarri, Paul Beisswenger, Weijing Cai, Aron M. Troen, Michal Schnaider Beeri

**Affiliations:** 1 The Nutrition and Brain Health Laboratory, The Institute of Biochemistry, Food and Nutrition Science, The Robert H. Smith Faculty of Agriculture Food and the Environment, The Hebrew University of Jerusalem, 7610001 Rehovot, Israel; aron.troen@mail.huji.ac.il; 2 The Joseph Sagol Neuroscience Center, Sheba Medical Center, 5265601 Tel Hashomer, Israel; thamar.Ganmore@sheba.health.gov.il (I.G.); Shelly.Shahar@mayo.edu (S.S.); moran281093@gmail.com (M.Z.); michal.beeri@mssm.edu (M.S.B.); 3 Memory Clinic, Sheba Medical Center, 5265601 Tel Hashomer, Israel; 4 Sheba Medical Center, Neurology Department, 5265601 Tel Hashomer, Israel; 5 Sackler Faculty of Medicine, Tel Aviv University, 6997801 Tel Aviv, Israel; 6 Department of Neurology, Mayo Clinic, Rochester, MN 55905, USA; 7 Icahn School of Medicine at Mount Sinai, New York, NY 10029, USA; jaime.uribarri@mountsinai.org (J.U.); weijing.cai@mssm.edu (W.C.); 8 PreventAGE Healthcare, Lebanon, NH 03766, USA; pjb@preventagehealthcare.com

**Keywords:** type 2 diabetes, advanced glycation end-products, diet, randomized controlled trial, adherence

## Abstract

Introduction: High serum concentrations of advanced glycation end-products (AGEs) in older adults and diabetics are associated with an increased risk of cognitive impairment. The aim of this pilot study was to assess the feasibility of long-term adherence to a dietary intervention designed to decrease intake and exposure to circulating AGEs among older adults with type 2 diabetes. Methods: Herein, 75 participants were randomized to either a standard of care (SOC) control arm or to an intervention arm receiving instruction on reducing dietary AGEs intake. The primary outcome was a change in serum AGEs at the end of the intervention. Secondary and exploratory outcomes included adherence to diet and its association with circulating AGEs. Cognitive function and brain imaging were also assessed but were out of the scope of this article (ClinicalTrials.gov Identifier: NCT02739971). Results: The intervention resulted in a significant change over time in several serum AGEs compared to the SOC guidelines. Very high adherence (above 80%) to the AGE-lowering diet was associated with a greater reduction in serum AGEs levels. There were no significant differences between the two arms in any other metabolic markers. Conclusions: A long-term dietary intervention to reduce circulating AGEs is feasible in older adults with type 2 diabetes, especially in those who are highly adherent to the AGE-lowering diet.

## 1. Introduction

Advanced glycation end-products (AGEs) are a class of chemical compound that are created by the non-enzymatic reaction between reducing sugars and free amino groups. This reaction occurs endogenously in the body during the course of normal cellular metabolism [1,2]. AGEs tend to be more abundant in the tissue of older humans rather than younger humans [3], as well as under conditions where the precursors for the reaction, such as circulating glucose, are in excess [1,2]. Thus, older adults with type 2 diabetes (T2D) tend to have higher levels of systemic AGEs.

In addition to the endogenous creation of AGEs, exogenous sources such as pre-formed AGEs from food contribute significantly to the body’s pool of AGEs. AGEs exist in most foods, but they are particularly abundant in animal-derived foods. Exposure to high temperatures (i.e., grilling, broiling, roasting, searing, and frying) and long cooking times can greatly increase the amount of AGEs in food [4,5].

AGEs affect cellular functions by binding to the receptor for advanced glycationend-products (RAGE), resulting in the activation of inflammatory and oxidative stress cascades [2,6]. High AGE levels are associated with a wide range of chronic diseases, such as T2D [7], atherosclerosis [8], ocular diseases [9], cancer [10], and kidney disease [11]. Increasing evidence also suggests the involvement of AGEs in neurodegenerative disorders [12], especially Alzheimer’s disease (AD).

T2D has been consistently shown to increase the risk ofAD [13]; diabetic individuals have relatively high levels of AGEs, in part due to high circulating glucose levels. In turn, AGEs have been shown to cross-link beta amyloid [14], the hallmark pathology of AD. Thus, increased chronic exposure to AGEs may provide a mechanistic explanation for the higherrisk of AD experienced by diabetic adults compared to non-diabetic older adults. If so, then achieving a lasting reduction of circulating AGEs by reducing dietary intake should reduce the associated risk or severity of disease. Current dietary guidelines for people with diabetes are designed to improve glycemic control but are not specifically designed to decrease AGEs. Short-term clinical trials have shown that middle-aged patients with T2D [15] and chronic kidney disease [16] can significantly reduce their circulating AGEs concentrations and improve biomarkers of disease severity (e.g., less insulin resistance in diabetes) by changing their cooking methods that reduce their dietary AGE intake [17]. Such encouraging findings underscore the importance of rigorously evaluating whether reducing dietary AGE intake can reduce cognitive risk. However, before this can be definitively tested in a randomized controlled clinical trial, it must be shown that such dietary changes will be acceptable and effective in older adults with diabetes, who have a high dementia risk, but may find it difficult to adapt to the necessary dietary changes, and who may show a different biological response to intervention.Therefore, we designed this pilot study to assess whether a six month intervention to decrease dietary AGEs would improve serum AGE levels relative to the dietary standard of care for older adults with T2D based on the American Diabetes Association (ADA) dietary recommendations [18].

## 2. Materials and Methods

The study was designed according to the CONSORT criteria extension to pilot trials [19] and was approved by the Helsinki Committee of the Sheba Medical Center, Israel (2206-15-SMC). All participants gave signed informed consent. The study was registered at www.clinicaltrials.gov (NCT02739971).

### 2.1. Screening

Minimum inclusion criteria to participate in the study included being age > 65, a diagnosis ofT2Dand subjective memory complaints. T2D was defined by the use of medications for T2D, health records indicating a diagnosis of T2D, or tworecent routine blood tests indicating fasting glucose above 126 mg/dl. Candidates with any existing neurological condition that can affect cognition were excluded (e.g., dementia, Parkinson’s disease, schizophrenia, traumatic head injury, etc.). All participants were required to have a family member as an informant. The study was advertised using mass mailing to recruit volunteers from among people who had previously agreed to receive health-related emails and by advertising through websites and social media forums for clinical trials, dementia, and diabetes. Interested candidates were contacted by telephone to ascertain age and T2D status and were invited to a face-to-face screening visit where cognition and nutritional status were assessed. Participants were eligible if they met the following criteria at the screening visit: a clinical dementia rating scale (CDR) <1 (i.e., normal cognition or questionable dementia, but no frank dementia) [20], Montreal Cognitive Assessment (MoCA) >18 (no dementia) [21], and geriatric depression scale (GDS) <6 (no clinical depression) [22]. In addition, a short dietary questionnaire was administered to assess AGEs intake using a translated version of a validated 7-day questionnaire with comparable food items that are frequently consumed in Israel and are rich in AGEs (e.g., meat, fish, poultry, cheese, egg yolk, fats, fast foods, and convenience breakfast and snack foods) [16]. Each item wasassigned an AGE equivalent based on a database of ~560 food AGE values [5]. Habitual AGE intake wasestimated from the frequency at which these items were reportedly consumed during the past week, after taking into account the portion sizes and the cooking methods (i.e., boiling, roasting, broiling/grilling, frying, or canned). The results wereexpressed as AGEs Eq/day, where 1 AGE equivalent = 1000 kilounits). To increase the likelihood of achieving a significant reduction of dietary AGEs intake, we followed a protocol used in similar trials whereby only participants with estimated AGEs intakes greater than 13 kU/day were invited to participate in the study [3].

### 2.2. Intervention

Upon enrollment, participants were assigned by a computerized random sequence generator to follow one of two diets for 6 months. The standard of care (SOC) arm was instructed to follow dietary guidelines for achieving good glycemic control [18]. The AGE-lowering arm received additional instruction on reducing dietary AGE intake. At the baseline visit, participants from both arms were provided dietary guidance by a registered dietitian. The main focus of the guidelines for good glycemic control for both arms was the intake of carbohydrates, including information on serving sizes, counting, and quality. Recommendations were given for carbohydrate intake from vegetables, fruits, whole grains, legumes, and low-fat dairy products while reducing the consumption of refined sugars. In addition, participants were advised to choose food items rich in mono and poly unsaturated fatty acids compared to saturated and trans fatty acid, as well as to consume lean meat, low sodium, and low processed food items. Other guidelines for good glycemic control were individualized based on the participants’ metabolic and nutritional needs, habits, preferences, willingness, and ability to make behavioral changes.

Participants were asked to follow the instructions of the study for 6 months and to avoid any new diet or life-style program during this period. Those assigned the AGE-lowering arm were provided the same guidance on glycemic control, but in addition were taught how to reduce their dietary AGEs intake by modifying the cooking methods and reducing cooking time and temperature without changing the nutrient composition of their food). They were advised to boil, poach, stew, or steam and to avoid frying, baking, or grilling anyanimal-derived products [5]. All participants also received the guidelines in writing. Informants were asked to join the nutritional counseling in case of a shared house with the participant. If the informant could not attend, the dietitian called and instructed him or her by phone. All participants in both arms were called once a week to ascertain and encourage adherence to their diet. Participants from the SOC arm were asked general questions about their nutrition during the week (e.g., “did you manage to follow the guidelines for your diabetes?”). Participants from the AGE-lowering arm were also asked specifically about their attempts to maintain low AGE intake, asking whether and how many times they cooked using methods that produce higher AGEs since the last call. If a participant reported consuming food items rich in AGEs, the dietitian repeated the instructions to lower AGEs by cooking modification of animal products to both the participant and the informant. Each follow-up call included personalized questions related to prior calls regarding personal challenges (such as preventing hypoglycemia or eating on holidays and special events) and progress (such as self-monitoring of fasting glucose). A summary of each telephone call was registered by the study dietitian including the date of the call and the main issues that arose. Failure to reach the participant was also registered.

### 2.3. Operational Measurement of Adherence with the Diet

To minimize potential bias, the study dietitian categorized participants’ self-reported adherence upon completion of the study but before serum AGEs results were available and before analyzing any outcomes. Each phone call scored 0 for a negative answer and 1 for a positive answer to the following questions:

1. Did the participant report fully maintaining the guidelines for good glycemic control since the previous call (yes/no for all participants)? As carbohydrates intake is a key strategy in achieving glycemic control [18], the answer for maintaining guidelines for good glycemic control was focused on the intake of food items from this group.
2. Did the participant report using any cooking methods that they wereadvised to avoid for animal derived food items since the previous call? (yes/no for AGE-lowering arm only).

Scores (0/1) obtained from each telephone call were summed for two adherence scales: A T2D adherence scale for all participants and a low AGEs adherence scale for the AGE-lowering arm only. The total score for each scale was divided by the average number of calls and was presented in percentage of “positive answers” reflecting adherence to the guidelines. A 1–5 Likert scale with the following categorization was built: A score of 1 represented participants who maintained the dietary instructions above 80% of phones call (“very high adherence”), a score of 2 represented those who complied with the diet between 60–80% of the calls (good adherence), and a score of 3 referred to adherence of between 40–60% (“partial adherence”). For adherence lower than 40%, participants were asked if they wished to continue participating in the study. A score of 4 represented those compliant below 40% of the calls but with a desire to continue participating in the study(“lack of adherence but desire to continue”), and finally, a score of 5 referred to those who had below 40% adherence and who wished to withdraw from the study (“lack of adherence and desire to withdraw”). If the total number of phone calls with the participant was below the 5th quintiles (=less than 14 phone call), another point was added to the final score of the adherence scale, as not answering phone reflect low adherence.

### 2.4. Assessments, Outcomes, and Measures

Identical assessments were repeated at baseline and post-intervention (after 6 months). A fasting blood sample was drawn to analyze markers related to diabetes (glucose, HbA1c, insulin), lipid profile (triglycerides [TG], high-density lipoprotein [HDL], low-density lipoprotein [LDL], total cholesterol), kidney function (Urea, Creatinine), the inflammatory marker C reactive protein (CRP), and a complete blood count. The Homeostatic Model Assessment of Insulin Resistance (HOMA-IR) was calculated according to the following equation: insulin (mu/l) Xglucose (mg/dl)/405 [23]. HOMA-IR was not calculated for participants using insulin (N = 18). Creatinine clearance was calculated using the Cockcroft–Gault equation: ((140−age) Xweight)/72XCreatinine (mg/dl) X 0.85 for females [24]. Blood was centrifuged and serum was separated and stored at −80 °C for subsequent AGEs analysis.

### 2.5. Serum AGEs Analysis

AGEs were measured in a blinded fashion by liquid chromatography–mass spectrometry (LC-MS) using an Agilent model 6490 Triple Quadrupole MS with a 1290 Rapid Resolution LC system and stable heavy isotope substituted internal standards, as described elsewhere [25] (PreventAGE Healthcare Technology, Lebanon, NH, USA). Briefly, duplicate serum samples were centrifuged through a 10-K cutoff Amicon filter. The filtrate contains free AGEs and peptides of various sizes, as well as the analytical method measures the free products. AGEs were separated using a Waters X-select HSS T3 column (2.5 mm, 2.1 3 150 mm) with a mobile phase gradient of methanol and water with 0.20% heptafluorobutyric acid. Five dicarbonyl-derived AGEs were assessed: Ne-carboxymethyl lysine (CML), Ne-carboxyethyl lysine (CEL), glyoxalhydroimidazolone (G-H1), methylglyoxalhydroimidazolone (MG-H1), and 3-deoxyglucosone hydroimidazolone (3DG-H1) [25].

### 2.6. Nutritional Assessment

General habitual dietary quality and nutritional intake was assessed with a validated Hebrew food frequency questionnaire (FFQ), which was specifically designed to assess nutrition in older adultsand is commonly used in this population [26]. The questionnaire includes 126 items and was administrated by the study dietitian. Dietary AGEs intake was assessed with the short AGEs questionnaire described above.

### 2.7. Other Measurements

Anthropometrics: Weight, height, and waist circumference (measured in centimeters at the level of the umbilicus) were assessed by the study dietitian. Participants wore their shoes and clothing to avoid discomfort but were asked to leave only basic clothing during measurements. Demographic information: year of birth, years of education, profession, and family status. Lifestyle: physical activity was assessed using the Minnesota Leisure Time Physical Activity Questionnaire [27] and smoking habits were recorded.

### 2.8. Blinding

Although diet allocation could not be concealed from the participants and study dietitian, care was taken to blind all other research staff to the arm assignments, including the neuropsychologist, the MRI technician, the lab technician where the blood tests were analyzed, and the research assistant who entered the data.

### 2.9. Statistical Analysis

The primary analysis of the effect of the intervention used intent-to-treat (ITT) methods and secondary analyses used per-protocol (PP) methods. The ITT analysis included all randomized participants who finished 6 months of follow-up. For the PP analysis, participants were considered to be fully compliant if they were at least 80% adherent to their assigned diet as assessed by weekly phone interviews (categorized as “very high adherence” on the adherence scale).

Baseline demographic and clinical characteristics are presented descriptively as proportions or as means with standard deviations. Comparison between arms was analyzed by χ^2^ test for categorical outcomes, *t*-test for continuous outcomes distributed normally, and by Mann–Whitney tests for non-normal distributions.

Changes in serum AGEconcentrations from baseline to 6 months were analyzed with paired *t* test or Wilcoxon tests for within-arm changes and by 2 sided *t*-test or Mann–Whitney for the between-arms analysis. Alpha of 0.05 was used for significance levels. SPSS version 23 (IBM Corp., Armonk, NY, USA) was used forall analyses.

## 3. Results

### 3.1. Baseline Characteristics and Retention

In total, 75 eligible participants were randomized to enter the study. Baseline characteristics of arms randomized to the AGE-lowering and SOC diet are shown in Table 1. There were no statistically significant differences between the arms at baseline for any variable, indicating that randomization was successful. The mean age was 71.6 ± 4.07 and 74.7% were males. Participants’ baseline macronutrient consumption did not differ by arm. Further, 24% used insulin and the average HbA1C% of 6.67 ± 0.67 in the entire sample is indicative of a diabetic population with good control of their blood glucose.

During the 6 month follow-up period, only five participants withdrew, 3 from the SOC arm and 2 assigned to the AGE-lowering diet so that 70 (93%) had complete data for analysis at the 6 months follow-up visit. The average number of completed phone calls was 16.5 calls per patient, with no significant difference in call rates between the two arms (*p* = 0.12) indicating that the extent of patient-dietitian interactions did not differ by arm.

### 3.2. Effect of Intervention on Nutritional Intake

Energy intake (Kcal) declined in both arms by 15.5% on average with no difference between the 2 diet arms (*p* = 0.34). The relative proportion of macronutrients at baseline was similar between the 2 diet arms and remained the same at follow-up, as it was at baseline for each diet, indicating that the decline in energy reflected a decrease in the amount of food consumed rather than a shift in food preferences (Table 2). Estimated AGEs intake reduced significantly in both arms; however, when comparing the between-arm differences in the magnitude of the effect of each diet, the AGE-lowering arm achieved more than twice the reduction in AGEs intake than the controls (−11.8 kU/day vs. −4.6 kU/day, *p* < 0.01; Table 2). Dietary AGEs density was calculated by dividing estimated AGEs intake by the total energy intake. In contrast to the SOC diet, whose dietary AGEs density did not change significantly over the 6 months of the study, the AGE-lowering arm had a significant decline and this difference between the arms was significant (−0.004 kU/kcal vs. −0.0006 kU/kcal *p* value = 0.001; Table 2).

### 3.3. Effect of Intervention on Circulating AGEs

The effect of the intervention on the five serum AGEs (CML, CEL, G-H1, MG-H1, 3DG-H1) is shown in Table 3. The ITT analysis shows that in control participants, all of the measured AGEs tended to increase from the baseline to the follow-up, with statistically significant increases for G-H1 (*p* < 0.001) and MG-H1 (*p* = 0.015). In contrast, in the AGE-lowering arm, circulating AGE concentrations tended to be relatively stable with slight declines that were not statistically significant. Between-arm comparisons of the diet-induced change in AGE serum levels was statistically significant for CEL (The AGE-lowering arm declined by 10% compared to a 4% increase in the SOC arm; *p* = 0.044) and MG-H1 (8% decline in the AGE-lowering arm and 27% increase in the SOC arm; *p* = 0.034).

### 3.4. Effect of Intervention on Metabolic Status

Participants assigned to both the SOC diet and the AGE-lowering diet improved in several metabolic parameters (Table 4). In the SOC arm, insulin and HOMA-IR decreased significantly without significant differences in any other metabolic markers. In the AGE-lowering arm, body mass index (BMI) significantly decreased without significant changes toany other metabolic markers.

### 3.5. Per-Protocol Analysis of Adherence to the AGE-Lowering Diet on Study Outcomes

We further analyzed if the reduction of AGE biomarkers in the AGE-lowering arm wasassociated with their level of adherence. In total, 19participants (57%) were classified as “very highly adherent”, 10 (30.9%) had “good adherence”, 4 (12.1%) had “partial adherence”, and none of the participants were classified as having “low adherence”. Since the “partial adherence” category had a small number of participants, we analyzed it together with the “good adherence” category. On average, all serum AGEs declined in the very highly adherent group, while they increased in those classified as having only good or partial adherence. The differences in the very high vs. good and partially adherent groups were statistically significant for CML (−10.7% vs. +8.7% respectively; *p* = 0.035), 3DGH (−10.9% vs. +31.7% respectively; *p* = 0.021), and MG-H1 (−30.2% vs. +23.6% respectively; *p* = 0.022) (Table 5).

Similarly, when metabolic markers were analyzed by adherence, only the very highly adherent group experienced a significant decrease in BMI of 0.77 kg/m^2^ (*p* = 0.012). Estimated AGE, energy, protein, carbohydrate, and fat intake all declined significantly in both groups. However, the decrease in estimated AGE intake was significantly greater in the very highly adherent group compared to the good and partially adherent group (−14.64 kU/day in the very high adherence vs. −7.91 kU/day in the good and partially adherent group *p* = 0.005).Other metabolic biomarkers tended to show a greater improvement in the very highly adherent vs. the less adherent groups but this was not statistically significant: HOMA-IR (−1.38 ± 3.07 vs. −0.53 ± 1.99; *p* = 0.41, respectively) and creatinine clearance (+1.10 ± 14.47 vs. −4.11 ± 11.50; *p* = 0.27 respectively). No other metabolic or nutritional differences were observed.

## 4. Discussion

Our results show that dietary reduction of AGE intake in older diabetics can have the additional benefit of decreasing circulating AGE levels, beyond that which can be achieved by following the standard of care diet for good glycemic control. As expected, reductions in serum AGEs were more prominent in individuals who were highly adherent to the diet. We designed our intervention based on several earlier randomized controlled trials of dietary AGE reduction, which had demonstrated that limiting dietary AGE intake by modification of cooking methods could reduce circulating AGEs, specifically CML and MG, in healthy individuals [17], as well as in patients with metabolic syndrome [28], diabetes [15], and renal failure [16]. Moreover, those studies yielded a 23–35% reduction of CML and MG, and we observed a more modest reduction of 8–10% of serum CEL and MG-H1. Several considerations might account for the relatively modest reduction in AGEs concentrations that we achieved. First, we considered a priori that older adults with T2D may respond differently to dietary AGE-lowering than younger diabetics. The average age in the earlier clinical trials was 60–62 [15,29,30], whereas our participants were on average a decade older. AGE concentrations increase with age and diabetes, and renal clearance of AGE decreases with age [3]. Because the concentration of circulating AGEs reflects the balance of intake, endogenous production, enzymatic removal, and clearance, the relative contribution of dietary intake to overall circulating AGEs may be relatively less important than other factors in this population. 

Of interest, participants in the SOC arm showed a tendency for all serum AGEs to increase over time reaching statistical significance for G-H1 (+30%) and MG-H1 (+33%).Other trials with participants with the metabolic syndrome and diabetes have also documented that participants who didnot follow the AGE-lowering diet hada tendency to increase serum AGE levels [15,28]. Our results suggest that a higher reduction of AGE intake may be needed in order to have a biological response in circulating AGEs in this population. It is also important to note that we assessed serum AGE concentrations using mass spectrometry in contrast to most other AGE clinical trials that used ELISA. The two methods are different and results may not be comparable. 

In contrast to other trials, dietary AGE-lowering did not improve HOMA-IR in our population. This might simply reflect the relatively modest reduction of circulating AGEs in our study and the older age of our population. Of note, our average baseline HbA1c at 6.6% wasrelatively low for older adults with T2D, thus indicating good glycemic possibly that could explainthe lack of effect of the low AGE diet on metabolic markers. Moreover, 24% of our patients did not have assessment of HOMA because they were medicated with insulin and the number of participants might have not been large enough to demonstrate an effect of AGE change. This together with the high education level, low drop-out rate, and high adherence to diet may indicate selection bias towards a relatively well educated, diet-conscious, motivated, and resilient study population. 

Future clinical trials should include participants with more severe T2D as they may be amenable to a greater reduction in metabolic markers so the possible “floor effect” of good glycemic control found in our sample may be avoided. In addition, as reported in previous trials in participants with T2D [15,29], the levels of HbA1c were not improved following the intervention of AGEs reduction nor by the SOC diet. While this could be the result of the relatively stable population as discussed above, it indicates that the improvement in the circulating AGEs in the intervention arm is attributed primarily to the reduction of the dietary AGEs and not the glycation process. Lastly, we can notrule out the possibility of aninterviewer biasthatmay have led the participants to reporta lower AGE intake than what actually was eaten. In order to assess the scope of this bias, we aimed to analyze the validity of the participant’s reportsby assessing if the effect on circulating AGEs wasaffected by adherence. The greater improvement in circulating AGEs among those who reported greater adherence to the low AGE diet suggests that this bias was at least somewhat limited. The study dietitian spoke to participants approximately weekly with a structured questionnaire that reviewed the prior week’s eating habits in relation to the diet, providing suggestions to improve the diet for the following weeks. That allowed for a semi quantitative measurement of adherence to the diet. In the AGE-lowering arm, there were significant and robust declines in serum AGEs levels in the very high adherence subgroup (over 80%), which comprised 57% of the AGE-lowering arm, suggesting that a high proportion of individuals wereable and willing to commit to a low AGE diet closely supervised by a nutritionist. These results emphasize that maintaining adherence may be a crucial component of a future RCT study design. In contrast, in the subgroup with good/partial adherence, there was in fact an increase in serum AGE levels during the intervention, pointing out that such a diet should be carefully followed in order to reach a beneficial effect. The adherence scale that we constructed for this study may be useful for “predicting” the extent of circulating AGE reduction and might be tested for other nutritional biomarkers as well. In future trials, and in clinical practice, working with a quantitative scale during an intervention phase can help find participants that need closer support and monitoring by a dietitian to increase adherence associated with improved clinical biomarkers and outcomes.

Our study has several limitations including that by the nature of the design (explicit dietary interventions) neither the investigators nor the patients were blind to the intervention. The dropout rate in this clinical trial was relatively low (6.7%) and the two arms did not differ neither in number nor cause of dropout. Yet, it is possible that the relatively high adherence to the low-AGE diet was related to its novelty. The novelty of a dietary approach may increase adherence, at least in the short-term. We attempted to minimize this by providing tailored (rather than general) recommendations for T2D [18] for the participants in the SOC arm, which may have provided a sense of novelty in this arm as well. In addition, the sample size of our study might not have been large enough to demonstrate an effect of AGEs reduction on metabolic markers in older adults with T2D. 

The study has several strengths. The number and length of phone calls with the two arms was essentially identical limiting the placebo effect. In addition, the study used MS for the measurements of AGEs, which is more precise than ELISA used in the most RCT in the field. Lastly, our low dropout rates emphasize the feasibility of such programs in older adults with T2D.

## 5. Conclusions

The current study provides new evidence that dietary AGE restriction is feasible in older adults with T2D and that there are modest decreases in specific serum AGE levels resulting from a low AGE diet unrelated to changes in other metabolic markers. These decreases occur primarily in individuals who are highly adherent to the diet. Larger and longer-term studies are required to ascertain the health benefits of low AGEs diet in older adults with T2D. The ability of participants from a clinical trial setting to maintain such dietary guidelines for long term should also be tested.

## Figures and Tables

**Table 1:** Baseline characteristic (*n* = 75).

Variable	AGEsLowering Arm (*n =* 35)	SOC Arm (*n =* 40)	*p*
Age (years)	71.91 *±* 4.20	71.42 *±* 3.99	0.57
Gender *n* (%)	8 (22.9)	11 (27.5)	0.64
Female	27(77.1)	29 (72.5)	
Male			
Education (years)	13.89 *±* 2.88	14.93 *±* 2.62	0.10
Family history of dementia	23 (65.7)	24(60)	0.61
*n* (%)			
No family history			
Smoking *n* (%)	19 (54.3)	15 (37.5)	0.31
Never	14 (40)	23 (57.5)	
In the past	2 (5.7)	2 (5)	
Current			
Medication for T2D *n* (%)			0.32
All but insulin	29 (82.9)	29(72.5)	
Insulin included	6 (17.1)	9(22.5)	
Diet only	0	2(5)	
MoCA	23.14 *±* 2.18	23.25 *±* 2.23	0.80
GDS	2.37 *±* 1.71	2.53 *±* 1.66	0.69
ADL	99.43 *±* 2.35	99.75 *±* 1.10	0.84
Physical activity (METS/day)	148.75 *±* 130.92	118.63 *±* 115.61	0.22
Waist circumference (cm)	106.74 *±* 10.77	106.05 *±* 10.81	0.78
BMI (kg/m^2^)	30.02 *±* 4.28	29.55 *±* 3.58	0.75
CRP (mg/l)	4.79 *±* 5.28	3.48 *±* 4.22	0.10
Insulin (mU/l) (*n* = 57)^1^	15.24 *±* 10.97	15.11 *±* 9.41	0.64
HbA1C %	6.63 *±* 0.72	6.71 *±* 0.67	0.59
HOMA-IR (*n* = 57)^1^	5.04 *±* 4.12	4.53 *±* 2.35	0.81
Creatinine clearance(ml/min)	84.75 *±* 26.27	85.86 *±* 22.35	0.84
Estimated AGEs intake (kU/day)	23.25 *±* 6.48	22.14 *±* 6.50	0.46
Protein (g)	94.89 *±* 27.71	96.15 *±* 27.44	0.73
Fat (g)	83.21 *±* 25.08	82.70 *±* 30.78	0.62
Carbohydrates(g)	232.94 *±* 66.67	241.75 *±* 60.45	0.53
Energy (kcal)	2012.26 *±* 488.83	2059.03 *±* 553.68	0.86
Serum CML (nmole/L)	127.91 *±* 51.06	115.15 *±* 39.43	0.26
Serum 3DG (nmole/L)	376.51 *±* 202.44	377.92 *±* 232.56	0.84
Serum CEL (nmole/L)	104.61 *±* 42.04	93.15 *±* 38.07	0.18
Serum G-H1 (nmole/L)	12.08 *±* 5.21	10.46 *±* 2.70	0.12
Serum MG-H1 (nmole/L)	271.41 *±* 164.68	216.71 *±* 158.51	0.05

All values expressed as Mean ± SD. Abbreviations and explanation: AGEs: Advanced glycation end products; SOC; Standard of care; T2D: Type 2 diabetes; MoCA: Montreal cognitive assessment; GDS: Geriatric depression scale; ADL: Activity of daily living; BMI: Body max index; CRP: C reactive protein; HbA1C: glycated hemoglobin; HOMA-IR: Homeostatic model assessment of insulin resistance; CML: Ne-carboxymethyl lysine; 3DG-H1: 3-deoxyglucosone hydroimidazolone; CEL: Ne-carboxyethyl lysine; G-H1: glyoxalhydroimidazolone; MG-H1: methylglyoxalhydroimidazolone. ^1^ insulin mean and HOMA-IR include only participants who do not take insulin.

**Table 2:** Nutritional intake at baseline and follow-up.

	AGE-Lowering Arm (*n* = 33)				SOC Arm (*n* = 37)				
	Baseline	Follow-Up	Delta	*p* ^1^	Baseline	Follow-Up	Delta	*p* ^1^	*p* ^2^
Energy (kcal)	2020.74 ± 493.16	1648 ± 381.32	−372.74 ± 410.73	<0.001	2069.66 ± 574.35	1804.09 ± 503.20	−265.57 ± 511.03	<0.001	0.34
Protein (g)	95.13 ± 28.53	76.63 ± 19.84	−18.50 ± 21.80	<0.001	96.56 ± 27.99	86.95 ± 28.51	−9.60 ± 26.35	0.03	0.13
Fat (g)	83.94 ± 25.26	68.1 ± 18.05	−15.84 ± 25.20	<0.001	84.01 ± 31.38	71.46 ± 23.97	−12.54 ± 24.23	<0.001	0.57
Carbohydrates (g)	232.62 ± 67.73	193.02 ± 57.39	39.60 ± 51.38	<0.001	240.50 ± 61.57	211.57 ± 58.40	−28.92 ± 65.66	0.01	0.45
% Carbohydrates	45.9 ± 7.1	46.7 ± 7.3	0.8 ± 5.5	0.41	47.5 ± 6.1	47.4 ± 6.4	0.4 ± 4.8	0.62	0.74
% Protein	18.8 ± 3	18.6 ± 2.6	−0.2 ± 3.1	0.68	18.7 ± 2.8	19.2 ± 3.3	0.5 ± 3.1	0.29	0.30
% Fat	37.2 ± 5.7	37.5 ± 5.8	−0.2 ± 5.4	0.8	36 ± 5.1	35.3 ± 4.4	0.7 ± 4.6	0.33	0.66
Estimate dAGEs intake (kU/day)	23.51 ± 6.57	11.72 ± 4.98	−11.78 ± 7.09	<0.001	22.65 ± 6.49	18.06 ± 5.91	−4.58 ± 7.56	<0.001	<0.001
AGEs density ^3^ (kU/Kcal)	0.012 ± 0.003	0.0074 ± 0.003	−0.0047 ± 0.005	<0.001	0.011 ± 0.002	0.010 ± 0.003	−0.0006 ± 0.004	0.385	0.001

All values expressed as mean or % ± SD. Abbreviations and explanation: AGEs: Advanced glycation end products; SOC; Standard of care. *p* ^1^ change between baseline and 6 months visit within group; *p* ^2^ difference in mean change between AGE-lowering vs. SOC groups. ^3^ AGEs density was calculated as estimated dietary AGE intake divided by energy intake.

**Table 3:** Effect of intervention on serum AGEs concentration (ITT analysis).

	AGE-Lowering Arm (*n* = 33)				SOC Arm (*n* = 37)				
Measured AGEs (nmole/L)	Baseline	Follow-Up	Change (%)	*p* ^1^	Baseline	Follow-Up	Change (%)	*p* ^1^	*p* ^2^
CML	128.2 ± 52.5	124.8 ±45.4	−2.5 ± 31.5	0.6	112.5 ± 35.6	122.5 ± 52.2	+8.8 ± 39.6	0.1	0.5
3DGH	372.0 ± 207.3	397.2 ± 223.6	+6.7 ± 52.8	0.7	352.6 ± 169.9	372.9 ± 174.2	+5.7 ± 57.4	0.4	0.9
CEL	105.2 ± 43.1	94.6 ± 42.5	−9.9 ± 43.7	0.09	88.3 ± 29.8	91.9 ± 38.0	+3.9 ± 33.3	0.1	0.04
G-H1	12.0 ± 5.3	12.0 ± 5.7	+1.6 ± 4.1	0.9	10.2 ± 2.5	13.3 ± 7.7	+30.3 ± 68.6	<0.01	0.1
MG-H1	270.5 ± 169.5	248.8 ± 172.0	−8.0 ± 67.1	0.3	193.1 ± 92.7	265.7 ± 170.0	+37.3 ± 92.9	0.01	0.03

All values expressed as mean change ± SD. Abbreviations and explanation: AGEs: Advanced glycation end products; SOC; Standard of care; ITT: Intention to treat analysis; CML: Ne-carboxymethyl lysine; 3DGH: 3-deoxyglucosone hydroimidazolone; CEL: Ne-carboxyethyl lysine; G-H1: glyoxalhydroimidazolone; MG-H1: methylglyoxalhydroimidazolone. *p* ^1^ change between baseline and 6 months visit within arm; *p* ^2^ difference in mean change between AGE-lowering vs. SOC arms.

**Table 4:** Mean change in metabolic markers after 6 months (ITT).

	AGE-Lowering Arm (*n* = 33)				SOC Arm (*n* = 37)				
	Baseline	Follow-Up	Delta	*p* ^1^	Baseline	Follow-Up	Delta	*p* ^1^	*p* ^2^
Insulin (mU/L)	14.74 ± 10.86	12.44 ± 8.96	(*n* = 25) *	0.10	14.05 ± 5.90	11.61 ± 5.09	(*n* = 27) *	0.04	0.90
			2.28 ± 6.87				−2.09 ± 4.43		
HbA1C%	6.61 ± 0.68	6.58 ± 0.81	−0.03 ± 0.46	0.20	6.72 ± 0.66	6.71 ± 0.61	−0.01 ± 0.51	0.78	0.84
Glucose (mg/dL)	131.12 ± 23.31	125.06 ± 21.49	−6.06 ± 23.75	0.15	126.97 ± 33.90	121.54 ± 22.75	−5.43 ± 27.53	0.23	0.91
HOMA-IR	4.90 ± 4.14	3.97 ± 3.32	(*n* = 25) *	0.08	4.42 ± 1.9	3.44 ± 1.47	(*n* = 27) *	0.02	0.87
			−0.94 ± 2.55				−0.872 ± 1.74		
Creatinine Clearance (mL/min)	85.52 ± 26.93	84.08 ± 25.06	−1.18 ± 13.31	0.61	88.23 ± 18.52	85.55 ± 18.78	−2.17 ± 7.76	0.10	0.70
BMI (kg/m^2)^	30.05 ± 4.30	29.54 ± 4.25	−0.50 ± 1.11	0.02	29.36 ± 3.63	28.93 ± 3.77	−0.42 ± 1.38	0.05	0.94
Waist (cm)	107.03 ± 10.62	106.13 ± 9.08	−0.89 ± 5.31	0.17	105.70 ± 10.97	105.16 ± 11.27	−0.83 ± 5.42	0.22	0.74
Physical activity (METs/day)	150.53 ± 131.83	143.13 ± 107.75	−7.39 ± 94.96	0.62	118.43 ± 117.72	153.31 ± 139.57	34.88 ± 132.62	0.12	0.12

All values expressed as mean ± SD. Abbreviations and explanation: ITT: Intention to treat analysis; AGEs: Advanced glycation end products; SOC; Standard of care; HbA1C: glycated hemoglobin HOMA-IR: Homeostatic model assessment of insulin resistance; BMI: Body max index. * Insulin and HOMA-IR include only participants who do not use insulin. *p* ^1^ change between baseline and 6 months visit within arm; *p* ^2^ difference in mean change between AGE-lowering vs. SOC arms.

**Table 5:** Mean change in AGEs levels between baseline and 6 months follow-up by level of adherence to the dietary instructions (AGEs-lowering arm).

	Very High Adherence (*n* = 19)				Good/Partial Adherence (*n* = 14)				
Measured AGEs (nmole/L)	Baseline	Follow-Up	Change (%)	*p* ^1^	Baseline	Follow-Up	Change (%)	*p* ^1^	*p* ^2^
CML	131.68 ± 57.47	117.09 ± 42.03	−11.0 ± 25.0	0.15	123.58 ± 46.62	135.40 ± 49.28	+9.5 ± 37	0.35	0.035
CEL	109.45 ± 49.98	88.07 ± 42.90	−19.5 ± 43.8	0.04	99.43 ± 32.67	103.57 ± 41.99	+4.1 ± 40.4	0.7	0.11
3DGH	378.40 ± 235.55	337 ± 196.78	−10.9 ± 34.9	0.19	363.35 ± 169.79	478.90 ± 238.85	+31.8 ± 64.9	0.09	0.021
GH1	12.26 ± 6.55	11.28 ± 3.97	−7.9 ± 30.5	0.42	11.72 ± 3.33	13.11 ± 7.55	+11.7 ± 54.1	0.43	0.27
MG-H1	276.30 ± 198.21	192.82 ± 118.60	−30.2 ± 53.0	0.02	262.66 ± 127.54	324.78 ± 206.34	23.65 ± 75.4	0.27	0.022

All values expressed as mean change ± SD. Abbreviations and explanation: AGEs: Advanced glycation end products; CML: Ne-carboxymethyl lysine; 3DGH: 3-deoxyglucosone hydroimidazolone; CEL: Ne-carboxyethyl lysine; G-H1: glyoxalhydroimidazolone; MG-H1: methylglyoxalhydroimidazolone. *p* ^1^ change between baseline and 6 months visit within arm; *p* ^2^ difference in mean change between AGE-lowering vs. SOC arms.
